# Polyphenols-Loaded Sericin Self-Assembling Nanoparticles: A Slow-Release for Regeneration by Tissue-Resident Mesenchymal Stem/Stromal Cells

**DOI:** 10.3390/pharmaceutics12040381

**Published:** 2020-04-21

**Authors:** Giulia Orlandi, Elia Bari, Laura Catenacci, Milena Sorrenti, Lorena Segale, Silvio Faragò, Marzio Sorlini, Carla Renata Arciola, Maria Luisa Torre, Sara Perteghella

**Affiliations:** 1 Department of Drug Sciences, University of Pavia, Viale Taramelli 12, 27100 Pavia, Italy; giulia.orlandi@unipv.it (G.O.); elia.bari@unipv.it (E.B.); laura.catenacci@unipv.it (L.C.); milena.sorrenti@unipv.it (M.S.); sara.perteghella@unipv.it (S.P.); 2 Department of Pharmaceutical Sciences, University of Piemonte Orientale, Largo Donegani 2/3, 28100 Novara, Italy; lorena.segale@uniupo.it; 3 Silk Division, Innovhub, Stazioni Sperimentali per l’Industria, 20133 Milan, Italy; silvio.farago@mi.camcom.it; 4 Innovative Technologies Department, SUPSI, University of Applied Sciences and Arts of Southern Switzerland, Via Pobiette 11, 6928 Manno, Switzerland; marzio.sorlini@supsi.ch; 5 PharmaExceed srl, 27100 Pavia, Italy; 6 Laboratorio di Patologia delle Infezioni Associate all’Impianto, IRCCS Istituto ortopedico Rizzoli, Via di Barbiano 1/10, 40136 Bologna, Italy; carlarenata.arciola@ior.it; 7 Department of Experimental, Diagnostic and Specialty Medicine (DIMES), University of Bologna, Via San Giacomo 14, 40126 Bologna, Italy

**Keywords:** silk-sericin nanoparticles, proanthocyanidins, quercetin, epigallocatechin gallate, tissue regeneration, mesenchymal stem/stromal cells

## Abstract

Mesenchymal stem/stromal cells (MSCs) are a therapeutic target to promote tissue regeneration, mainly when oxidative stress-mediated damage is involved in disease pathogenesis. Here, slow-release silk sericin nanoparticles (SNPs) loaded with natural antioxidant polyphenols were developed to sustain regeneration by tissue-resident MSCs. SNPs were prepared by exploiting a self-assembly method with poloxamer and were loaded with proanthocyanidins (P), quercetin (Q) or epigallocatechin gallate (E). SNPs, with a diameter less than 150 nm, were able to encapsulate both hydrophilic (P and E) and hydrophobic (Q) drugs. A slow and controlled release was obtained from SNPs for all the actives in PBS, while in EtOH, Q and E showed a burst release but P did not. Kinetic models revealed lower diffusion of P than other biomolecules, probably due to the higher steric hindrance of P. The in vitro anti-oxidant, anti-elastase and anti-tyrosinase properties of SNPs were assessed: loading the P and E into SNPs preserved the in vitro biological activities whereas for Q, the anti-elastase activity was strongly improved. Moreover, all formulations promoted MSC metabolic activity over 72 h. Finally, SNPs exhibited a strong ability to protect MSCs from oxidative stress, which supports their potential use for regenerative purposes mediated by tissue-resident MSCs.

## 1. Introduction

The potential use of mesenchymal stem/stromal cells (MSCs) in tissue regeneration is based on their ability to produce a large variety of bioactive trophic factors that stimulate neighboring parenchymal cells to repair damaged tissues [1,2,3]. Therefore, MSCs are now considered as a therapeutic target to promote healing in many chronic and acute degenerative diseases, particularly when oxidative stress damage is involved in the pathogenic mechanisms. In fact, oxidative stress has detrimental effects on the longevity and metabolic functions of MSCs. It inhibits proliferation, increases senescence and ageing by down-regulating autophagy [4], and it inhibits MSC immunomodulation [2,5]. Flavonoids, which have strong antioxidant activity, could effectively protect MSCs from oxidative damage and, consequently, from senescence and ageing.

In this work, silk sericin nanoparticles (SNPs) were developed to target the delivery of proanthocyanidins (P), quercetin (Q) and epigallocatechin gallate (E) to MSCs. P, Q and E are flavonoids that belong to different subclasses. P are condensed tannins, which are abundantly present in flowers, fruits and seeds of various plants, where they act as a defense mechanism against pathogens and predators. Q is a flavanol, and it is primarily found in fruits and vegetables, while E is categorized as catechin and it is abundantly present in tea. Multiple biological effects have been attributed to P, Q and E, including antioxidant, anti-tyrosinase, anti-elastase, anti-inflammatory, antimicrobial and anti-cancer properties [6,7,8]. Notably, the anti-elastase activity of flavonoids may be helpful in slowing down the degradation of the elastin in tissue, which is generally caused by the excess of protease activity that follows tissue damage [9].

Silk sericin (SS) is a globular water-soluble protein synthesized in the labial gland of the silkworm *Bombyx mori*. SS has a molecular weight higher than 200 kDa and consists mainly of polar amino acids (78%), in particular serine and aspartic acid, while non-polar amino acids make up 22% of the protein. However, there are still significant differences in the amino acid composition according to the cocoon variety [10]. Notably, SS also contains flavonoids and carotenoids as impurities, which are responsible for its intrinsic antioxidant, anti-tyrosinase and anti-elastase activity [11]. In recent years, SS has been considered and managed as a waste product from the textile industry. Recently, however, SS biological properties have been exploited for biomedical and pharmaceutical purposes [12,13,14,15,16,17].

In the drug delivery field, SS has been employed, among other proteins, in the formulation of drug delivery systems in the form of both micro [18,19] and nanoparticles [14,20]. Protein-based nano-systems are being used to improve the cellular uptake and the body’s distribution of the drug, as well as to replace materials that are not biocompatible or have a negative impact on the environment. In this context, its excellent biocompatibility, controllable biodegradability, and non-immunogenicity make SS an ideal candidate for the formulation of drug delivery systems. Also, a recent review pointed out that thanks to their intrinsic biological activity, silk proteins, including SS, may be able to improve and support some of the effects of the active principle ingredient [14]. Unfortunately, nanoparticles based on SS “alone” cannot be obtained, due to its physicochemical instability, which is a result of its high water-solubility. To overcome this problem, several formulation strategies have been proposed, including conjugation, cross-linking or blending with other polymers [21,22,23], protein functionalization [24] or by employing particular techniques, such as the desolvation method [25] and electrospraying [26]. It has been previously shown that silk-based nanoparticles are effectively taken in by MSCs [27,28], however, efforts need to be made to guarantee the slow release of actives after MSC internalization. Indeed, one of the limitations to date for the use of nanoparticles in therapy is that in many cases there is a burst release of the drug after administration, which corresponds to the release of the drug fraction adsorbed on the external surface of the nanoparticle. Therefore, the drug is released before reaching its pharmacological target, namely, MSCs, which leads to lower activity [29]. In this context, we first developed slow-release SNP formulations that did not have this detrimental burst release effect; moreover, we investigated the kinetics of drug release to clarify the drug delivery mechanisms. Then, SNP biological activities were investigated in vitro in terms of its anti-oxidant, anti-elastase and anti-tyrosinase activity. Finally, in vitro studies were performed on tissue-resident MSCs that mediate regeneration. In particular, the potency of SNPs was assessed with respect to metabolic activity improvement and protection against oxidative stress damage.

## 2. Materials and Methods

### 2.1. Materials

Dimethyl sulfoxide (DMSO), ethanol (EtOH), hydrochloric acid (HCl) and hydrogen peroxide (H_2_O_2_) were purchased from Carlo Erba, Milan, Italy. Pluronic F-127 (Lutrol^®^ F127) was purchased from BASF, Ludwigshafen, Germany. 3-(4,5-dimethylthiazol-2-yl)-2,5-diphenyltetrazolium bromide (MTT), arbutin, 2,2-diphenyl-1-picrylhydrazyl (DPPH), E, *N*-Succinyl-Ala-Ala-Ala-*p*-Nitroanilide, pancreatic porcine elastase (PPE), polysorbate 20, Tris(hydroxymethyl)aminomethane (TRIS), tyrosinase, and Q were purchased from Sigma Aldrich, Milan, Italy. P was purchased from Indena, Milan, Italy.

### 2.2. Nanoparticle Preparation and Characterization

#### 2.2.1. Silk Sericin Extraction

*Bombyx mori* poly-hybrid cocoons were cut into pieces and degummed in an autoclave (Systec V-65, Wurttemberg, Germany) at 120 °C for 1 h (40 mL water/g of cocoons) [11]. The obtained SS solution was filtered using 70 μm cell strainers (Thermo Fisher Scientific, Milan, Italy) to eliminate larger impurities, frozen at −80 °C and freeze-dried (Modulyo^®^ Edwards Freeze dryer, Kingston, New York, NY, USA) at 8 × 10^−1^ mbar and −50 °C for 72 h. Finally, SS powder was stored at −20 °C until use.

#### 2.2.2. Nanoparticle Preparation

SNPs were prepared according to previously reported procedures with modifications [21]. Briefly, freeze-dried SS powder and Pluronic^®^ F-127 were dissolved in DMSO at a final concentrations of 0.5 and 2.5% (*w*/*v*), respectively. The active ingredient was added to the solution at a final concentration of 0.1% (*w*/*v*) and maintained under magnetic stirring at 37 °C until complete dissolution. Subsequently, the resultant solution mixture was added dropwise to deionized water under stirring, allowing the formation of SNPs by self-assembly. The obtained nanoparticle suspension was then dialyzed against deionized water using cellulose dialysis tubes (12–14 kDa, Thermo Fisher Scientific, Milan, Italy) for 72 h, sonicated for 1 h and centrifuged at 3000× *g* for 5 min (Thermo Scientific SL8 Centrifuge, Milan, Italy) to precipitate aggregates. The supernatant fraction was collected, frozen at −80 °C, and freeze-dried (Modulyo^®^ Edwards Freeze dryer, Kingston, New York, NY, USA) at 8 × 10^−1^ mbar and −50 °C for 72 h, and stored at room temperature until use. Overall, four formulations were considered, as reported in Table 1.

#### 2.2.3. Drug Loading, Production Yield and Encapsulation Efficiency Evaluation

SNP drug loading was evaluated by a spectrophotometer method (UV/VIS Spectrometer Lambda20, PerkinElmer, Wellesley, MA, USA) at 279, 275 and 373 nm for P, E and Q, respectively. Briefly, SNP-P and SNP-E were dissolved in deionized water plus HCl (0.1%, *v*/*v*) (1 and 2 mg/mL, respectively) while SNP-Q was dissolved in ethanol 96% (*v*/*v*) at the final concentration of 1 mg/mL, and maintained at mild magnetic stirring in the dark. The drug content was measured from standard calibration curves obtained by analyzing a concentration range of 10–80 µg/mL, R^2^ = 0.99 for P; 0.5–20 µg/mL for Q, R^2^ = 0.98; 5–50 µg/mL for E, R^2^ = 0.99. EtOH or water was considered as blank. The drug loading (% *w*/*w*) of each formulation was calculated from the ratio between the total drug content (extrapolated from the calibration curve) and the concentration of analyzed SNPs. Each measurement was performed in triplicate.

SNP production yield was calculated according to Equation (1) as follows:

Y (%) = (total weight of nanoparticles)/(weight of sericin + weight of drug + weight of poloxamer) × 100(1)

Encapsulation efficiency (EE%) was determined as the percentage ratio between the actual entrapped drug and the drug dissolved in the DMSO solution during SNP preparation.

#### 2.2.4. Nanoparticle Size Distribution

SNP size distribution was analyzed by nanoparticle tracking analysis (NTA) using NanoSight NS300 equipment (Malvern Panalytical, Grovewood Rd, WR14 1XZ, Great Malvern, Worcestershire, UK). SNPs were dispersed in water, vortexed, and sonicated for 5 min before carrying out NTA analyses. For each batch, five measurements of 90 s each were performed.

#### 2.2.5. Morphological Evaluation by Scanning Electron Microscopy (SEM)

SNP morphology was evaluated by SEM (MIRA3, Tescan, Brno, Czech Republic). Freeze-dried SNPs were carbon-sputter coated under argon before performing the morphological evaluation.

#### 2.2.6. Physical-Chemical Characterization

Thermal analysis, supported by Fourier-transform infrared spectroscopy (FT-IR), was used to characterize polymer, SS and drug bulks, as well as unloaded and drug-loaded SNPs. Temperature and enthalpy values were measured by differential scanning calorimetry (DSC) by a Mettler STAR^e^ system (Mettler Toledo, Novate Milanese, Milan, Italy) equipped with a DSC821^e^ module and an intracooler device for sub-ambient temperature analysis (Julabo FT 900). Samples in the range 1–3 mg were weighed (Mettler M3 Microbalance) and placed in sealed aluminum pans with pierced lids (β = 10 K min^−1^, nitrogen atmosphere (flow rate 50 mL min^−1^), −10/400 °C temperature range). The instrument was preventively calibrated with indium as a standard reference. Measurements were carried out at least in triplicate.

A Mettler STAR^e^ thermogravimetric analysis (TGA) system (Mettler Toledo, Novate Milanese, Milan, Italy) with simultaneous DSC (TGA/DSC1) was used to measure mass losses upon heating 2–3 mg samples in alumina crucibles with lids (β = 10 K min^−1^, nitrogen atmosphere (flow rate 50 mL min^−1^), 30/400 °C temperature range). Calibration procedure and triplicate measurements were applied, as for DSC above.

Mid-IR (650–4000 cm^−1^) spectra were recorded on powder samples using a Spectrum One FT-IR spectrophotometer (Perkin-Elmer, Wellesley, Minneapolis, MN, USA) equipped with a MIRacle™ ATR device (Pike Technologies, Madison, WI, USA) (resolution 4 cm^−1^).

#### 2.2.7. In Vitro Drug Release Test

The drug release from SNPs was evaluated by the dialysis technique according to the previously reported procedure in [30], with slight modifications. Experiments were repeated considering two different dissolution media: (i) EtOH/water in a 50/50 ratio for all the actives (P, Q and E) or (ii) PBS for P and E, and PBS + polysorbate 20 (6% *w*/*w*) for Q. Briefly, for each batch 200 mg of SNPs were suspended in 5 mL of dissolution media and put into a dialysis membrane (3.5 kDa MWCO, Thermo Fisher Scientific, Milan, Italy). Each dialysis tube was incubated in 50 mL of dissolution media and maintained under mild magnetic stirring at 37 °C. At each considered time point, an aliquot of release medium was collected and replaced with fresh medium to maintain sink conditions. The amount of released drug was determined by a spectrophotometric method (UV/VIS Spectrometer Lambda20, PerkinElmer, Wellesley, MA, USA) analyzing the release media at 279, 275 and 373 nm for P, E and Q, respectively. The drug concentration was extrapolated from a calibration curve previously prepared (P 5–80 µg/mL, R^2^ = 0.99; Q 0.5–15 µg/mL, R^2^ = 0.99; E 5–30 µg/mL, R^2^ = 0.98). Each experiment was performed in triplicate. The cumulative amount of released drug was calculated as a percentage using the following Equation (2):

Cumulative amount of drug released (%) = C_i_/C_0_ × 100 (2)

where C_i_ is the amount of the drug released at a definite time interval and C_0_ is the loaded drug amount.

#### 2.2.8. Drug Release Kinetic Study

The in vitro drug release data was interpolated using different kinetic models, as reported below.

Higuchi

*F*(*t*) = *k* × *t*^0.5^ (3)

*F*(*t*) = 100 × (1 − C × exp^(−*k*×*t*)^) (4)

where *F*(*t*) is the amount of drug dissolved at time *t* and *k* is the release constant. Equation (4) was reproduced from (Equation (2.12) from [31]).

Peppas–Sahlin

*F*(*t*) = *k*_1_ × *t^m^* + *k*_2_ × *t*^(2×*m*)^ (5)

where *F*(*t*) is the amount of drug dissolved at time *t*, *k*_1_ is the diffusion constant, *k*_2_ is the erosion constant and *m* is the diffusional exponent, indicative of the drug release mechanism.

Ritger–Peppas

*F*(*t*) = *k* × *t^n^* (6)

where *F*(*t*) is the amount of drug dissolved at time *t*, *k* is the release constant, and *n* is the release exponent, indicative of the drug release mechanism.

Zero-order

*F*(*t*) = *k* × *t* (7)

where *F*(*t*) is the amount of drug released in time *t*, and *k* is the release constant.

Korsmeyer–Peppas

*F*(*t*) = *k_KP_* × *t^n^* × Q_0_ (8)

where *F*(*t*) is the amount of drug released at time *t*, *k_KP_* is the release constant, *n* is the release exponent, indicative of the drug release mechanism, and Q_0_ is the initial amount of drug.

### 2.3. In Vitro Biological Activity

#### 2.3.1. ROS-Scavenging Activity

The ROS-scavenging activity of SNPs was evaluated by the DPPH colorimetric assay as previously reported [13,18,32,33] with slight modifications. Briefly, each SNP formulation was tested considering different final concentrations in the final reaction mix (15, 10, 5 and 2.5 mg/mL) and their free drug equivalent concentration calculated from the loading data. SNPs were left 24 h under magnetic stirring at room temperature to allow the release of actives. Either 120 µL of SNP or free drug samples were added to 1080 µL of a DPPH solution (0.0056% *w*/*v* in methanol 70% *v*/*v*). The reaction mix was incubated in the dark at room temperature, centrifuged at 3000× *g* for 5 min and subjected to spectrophotometric analysis (Synergy HT, BioTek, Swindon, UK) at 515 nm. A reaction mix without sample was used as a negative control. ROS-scavenging activity percentage was calculated according to the following formula:

ROS-scavenging activity (%) = [(A_ctr_ − A_samp_)/A_ctr_] × 100 (9)

where A_ctr_ is the absorbance of the negative control and A_samp_ is the absorbance of the sample. Each experiment was performed in triplicate.

#### 2.3.2. Anti-Elastase Activity

The anti-elastase activity was investigated for each SNP formulation considering different final concentrations in the final reaction mix (15, 10, 5 and 2.5 mg/mL) and their free drug equivalent concentration calculated from the loading data. SNPs were left for 24 h under magnetic stirring at room temperature to allow the release of actives. Then, the procedure reported by Bari and colleagues was employed [34]. Briefly, PPE was solubilized in phosphate buffer pH 6.8 (0.5 IU/mL). The substrate *N*-Succinyl-Ala-Ala-Ala-*p*-Nitroanilide was diluted in TRIS buffer to obtain a final concentration of 0.41 mM. Each sample was incubated for 20 min with the enzyme, and subsequently, the substrate was added. The kinetic reaction was monitored by spectrophotometric analysis (Synergy HT, BioTek, Swindon, UK) at 410 nm for 35 min (one measurement each minute). The reaction mix in the absence of sample was used as a negative control, while E was considered as a positive control (concentration tested: 7.2 mg/mL). All analyses were performed in triplicate, and the results are reported as the anti-elastase activity percentage, calculated using the following equation:

Anti-elastase activity (%) = [(A_ctr_ − A_samp_)/A_ctr_] × 100 (10)

where A_ctr_ is the absorbance of the negative control and A_samp_ is the absorbance of the sample.

#### 2.3.3. Anti-Tyrosinase Activity

The anti-tyrosinase activity of SNPs was evaluated by spectrophotometric analysis of the kinetic reaction between the enzyme tyrosinase and L-tyrosine substrate. For each SNP formulation, the anti-tyrosinase activity was tested considering different final concentrations in the final reaction mix (15, 10, 5 and 2.5 mg/mL) and their free drug equivalent concentration calculated from the loading data. SNPs were left for 24 h under magnetic stirring at room temperature to allow the release of actives. The enzyme tyrosinase was solubilized in phosphate buffer pH 6.8 to obtain a final concentration of 500 IU/mL. The tyrosinase solution was pre-incubated for 10 min with each sample and, consequently, the L-tyrosine substrate was added to the reaction mix. The enzymatic reaction was spectrophotometrically analyzed (Synergy HT, BioTek, Swindon, UK) at 480 nm for 35 min (one measurement each minute). The reaction mix without sample was used as a negative control, while arbutin was considered as a positive control (concentration tested: 2.5 mg/mL). All analyses were conducted in triplicate, and the results are reported as anti-tyrosinase activity percentage, calculated using the formula:

Anti-tyrosinase activity (%) = [(A_ctr_ − A_samp_)/A_ctr_] × 100 (11)

where A_ctr_ is the absorbance of the negative control and A_samp_ is the absorbance of the sample.

#### 2.3.4. Cell Metabolic Activity Evaluation

The cytocompatibility and proliferation ability of SNP formulations were evaluated on human adipose mesenchymal stem cells (MSCs). MSCs were isolated from adipose tissue samples, as reported in the Supplementary Materials. MSCs fulfilled adherence to the International Society for Cellular Therapy criteria [35]. MSCs were seeded in a 96-well plate (5000 cells/cm^2^) and cultured with DMEM/F12 supplemented with 10% (*v*/*v*) fetal bovine serum (FBS), 100 U/mL penicillin, 100 µg/mL streptomycin, 0.25 µg/mL amphotericin, 4 mM glutamine, 1 mM sodium pyruvate. After 24 h, the supernatants were replaced with 100 µL of culture medium (not supplemented with FBS) containing SNPs at the final concentrations of 0.8, 0.4, and 0.2 mg/mL, or their free drug equivalent concentrations calculated from the loading data. SNPs were left for 24 h under magnetic stirring at room temperature to allow the release of actives. After 24, 48 and 72 h of incubation, supernatants were discarded, cells were then washed with PBS, and 100 μL of MTT solution (0.5 mg/mL) was added to each well. After three hours of incubation, the MTT solution was removed, and 100 μL of DMSO was added. Untreated cells were considered as control (100% of metabolic activity). The absorbance was measured by a microplate reader (Synergy HT, BioTek, Swindon, UK) at 570 nm and 670 nm (reference wavelength). Each condition was tested in triplicate, and the percentage of cell metabolic activity was calculated as follows:

Cell metabolic activity (%) = 100 × (Abs_sample_/Abs_ctr_) (12)

where Abs_sample_ is the mean value of the measured absorbance of the tested samples, and Abs_ctr_ is the mean value of the measured absorbance of cells not incubated with SNPs or free drug. All experiments were performed in triplicate.

#### 2.3.5. Oxidative Stress Protection Test

SNPs have been tested in terms of cell protection from the oxidative stress damage. MSCs were seeded in 96-well plate (5000 cells/cm^2^) and treated with 100 µL of culture medium containing SNPs at the final concentrations of 0.8, 0.4 and 0.2 mg/mL or their free drug equivalent concentration calculated from the loading data. SNPs were left 24 h under magnetic stirring at room temperature to allow the release of actives. After 24 h, the media was discarded, and 100 µL of hydrogen peroxide (1.5 mM) solution were added to each well. Cells not incubated with H_2_O_2_ were considered as control. After 24 h, for the cells incubated with or without H_2_O_2_, an MTT test was performed to evaluate the cellular metabolic activity, calculated as previously reported in Section 2.3.4. All experiments were performed in triplicate.

### 2.4. Statistical Analysis

Raw data were processed by STATGRAPHICS XVII (Statpoint Technologies, Inc., Warrenton, VA, USA). For data with a normal distribution, a linear generalized analysis of variance model (ANOVA) was used and combined with Fisher’s least significant difference (LSD) procedure to evaluate the differences between the groups. In detail, drug loading results were analyzed considering the batch as a fixed factor and the drug loading as the response variable. NTA analysis results were analyzed considering the formulation as a fixed factor and mean diameter, mode, d_10_, d_50_ and d_90_ as the response variables. Release data were processed considering the amount of active released as the response variable and the formulation and times as the fixed factors. Cumulative drug release data was interpolated and curve kinetic parameters for each model were determined using Graph-Pad Prism software version 8.0.1 (GraphPad Software 2365 Northside Dr. Suite 560 San Diego, CA 92108, USA). The ROS-scavenging data were elaborated considering the sample as a fixed factor, the concentration as a covariate and the ROS-scavenging activity (%) as the response variable. Anti-elastase and anti-tyrosinase raw data were elaborated considering the sample and time as fixed factors, the concentration as a covariate and the activity % as response variables. The enzymatic kinetics of anti-tyrosinase and anti-elastase activity were elaborated with Michaelis-Menten model kinetics *y* = (*V_max_* × *x*)/(*K_m_* + *x*), where *y* is the absorbance at time *x*, *K_m_* is the moment in which the activity is equal to half the maximum and *V_max_* is the maximum speed of the enzyme [11]. Graph-Pad Prism software version 8.0.1 (GraphPad Software 2365 Northside Dr. Suite 560 San Diego, CA 92108, USA) was used to calculate the curve parameters. For each curve, Vmax and Km were analyzed with an ANCOVA model, considering the sample as a fixed factor and the sample concentration as a covariate. The differences between the groups were analyzed with the LSD test for multiple comparisons. Proliferation data were analyzed considering the sample concentration and time as fixed factors, the concentration as a covariate and the cell metabolic activity (%) as the response variable. Oxidative stress data were elaborated considering H_2_O_2_ concentrations (0 or 1.5 mM) and the sample as fixed factors, the sample concentrations as a covariate and the cell metabolic activity (%) as the response variable. For all the analyses, the statistical significance was set up at *p* < 0.05. Unless otherwise specified, data are expressed as mean ± standard deviation.

## 3. Results and Discussion

SS is a promising biocompatible and bioactive material and it was selected here for the preparation of nanoparticles, and to obtain the slow release of naturally-derived biologically active substances. Specifically, SNPs were prepared by exploiting a self-assembly method with poloxamer; three different active ingredients were loaded into SNPs: P, Q and E. For each formulation, three different batches were prepared.

**Figure 1:**
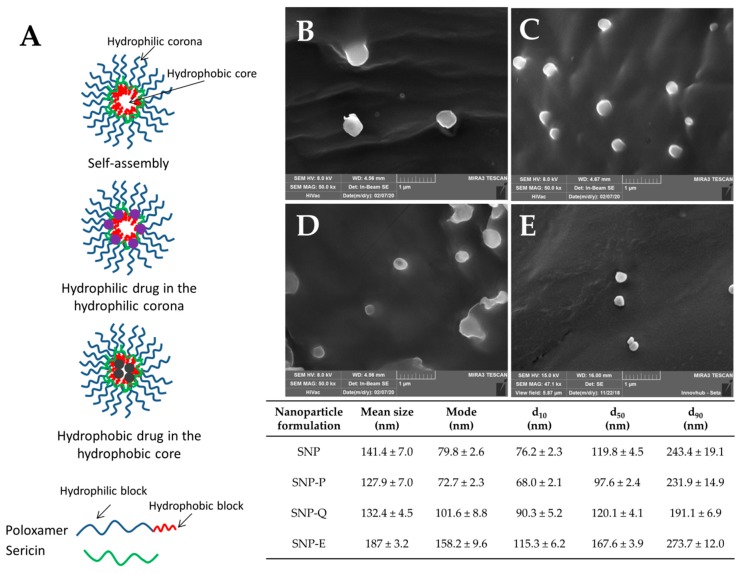
(**A**) Proposed structure for silk sericin nanoparticles (SNPs). SEM images and particle size distribution of SNP-P (**B**), SNP-Q (**C**), SNP-E (**D**) and SNP (**E**). Scale bar 1 µm. Nanoparticle tracking analysis (NTA) results are reported as mean, mode, d_10_, d_50_ and d_90_ values, all of them ± standard error, *n* = 5. Statistical analysis revealed no significant differences in particle size and particle size distribution among the different formulations (*p* > 0.05).

The process yield (%) in the SNP preparations ranged from 60.9 ± 0.46% for SNP-Q to 63.8 ± 4.25% for SNP-P. These values are compatible with the small batch sizes (about 1.5 g) obtained in our lab-scale process. The SNPs were able to encapsulate both hydrophilic (P and E) and hydrophobic (Q) drugs (Table 2). Considering the proposed structure of SNPs (Figure 1A), hydrophobic drugs should reside in the inner core of the micellar structure, while a hydrophilic drug is expected to reside within the corona, which is relatively hydrophilic. It can be supposed that the interaction between hydrophilic actives and the hydrophilic corona is due to the formation of ionic interactions or hydrogen bonds, while hydrophobic actives are entrapped in the hydrophobic core by hydrophobic effects and Van der Waals forces [36]. The statistical analysis revealed no significant differences in terms of drug loading and encapsulation efficiency among different batches of the same active ingredient (*p* > 0.05), indicating proper standardization of the final product (see Figure S1 reported in the Supplementary Materials). However, significant differences were found between the different actives considered (*p* < 0.001). In detail, encapsulation efficiency (EE) values were higher for the hydrophilic drugs P and E than for the hydrophobic Q (Table 2). As previously reported [37,38], drug-core compatibility is one of the most critical factors that may influence the loading efficiency of SNPs. Sunoqrot and colleagues [39] reported that, according to the Flory–Huggins interaction parameter, ideally, the highest drug-core compatibility is achieved when the water solubility values of the drug and hydrophobic core are equal. In our case, the different solubility of poloxamer and Q (50 mg/mL vs. 2 mg/mL in water at 25 °C) may have affected the drug loading and explain the low encapsulation efficiency.

All nanoparticles were nanometric in size. SNP-E showed a higher average diameter (201.4 ± 15.15 nm) compared to SNP-P (141.2 ± 15.15 nm) and SNP-Q (137.4 ± 12.37 nm) (mean value ± SE, *n* = 5). Statistical analysis revealed that the encapsulation of active substances did not significantly influence the particle size and size distribution (*p* > 0.05). These results were confirmed by SEM morphological investigation: all SNP formulations showed a nanometric size and spherical shape with a smooth surface (Figure 1B–E).

**Figure 2:**
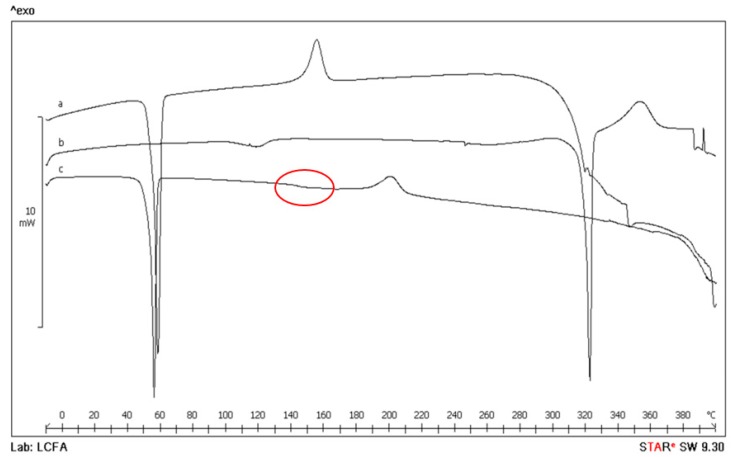
Differential scanning calorimetry (DSC) thermal profile of Lutrol^®^ F127 (curve a), Q (curve b) and SNP-Q (curve c).

Thermal analysis, supported by FT-IR spectroscopy, was used to characterize polymer, SS and free drugs, as well as unloaded and drug-loaded SNPs. In Figure 2, for example, the thermal profiles of SNP-Q are reported. The DSC profile of Lutrol^®^ F127 (curve a) shows an endothermic effect at T_peak_ = 58.1 ± 0.8 °C (T_onset_ = 55.2 ± 0.4 °C, ΔH_m_ = 110 ± 1 J g^−1^) due to melting, followed by an exothermic effect peaking at about 155 °C due to the melt decomposition [40]. Q is a crystalline compound showing a DSC thermal profile with an initial broad effect at about 118 °C due to dehydration (as confirmed by TGA mass loss of 0.8 ± 0.5%, curve not reported), and a second endothermic effect at T_peak_ = 321.9 ± 0.2 °C (T_onset_ = 319.1 ± 0.5 °C, ΔH_m_ = 168 ± 3 J g^−1^) corresponding to melting, followed by exothermic decomposition at about 350 °C (curve b). In the trace of loaded SNPs (curve c), the thermal effect due to the polymer melting is still present with thermal and enthalpic parameters slightly lower at T_peak_ = 55.8 ± 0.6 °C (T_onset_ = 53.5 ± 0.5 °C, ΔH_m_ = 99 ± 2 J g^−1^) due to the presence of the other ingredients in the system as impurities. At about 150 °C, an effect due to the glassy-rubbery transition (T_midpoint_ = 146 ± 2 °C, red circle) attributable to the random coil domains present in the amorphous region of the SS protein structure and also, the exothermic peak at 200 ± 2 °C, which can be attributed to the transition from the random coil to β-structure of SS, are visible. These effects in the DSC curve of loaded SNPs confirm the presence of the protein in nanoparticles. No effect due to the presence of the active is evident, suggesting that probably Q is molecularly dispersed in the polymer. Also, it is probably not possible to detect the presence of Q in the final system due to the superimposition of drug melting with the polymer decomposition. The DSC profile of SNP-P is not reported because of its flat profile due to the prevalent amorphous nature of the active. In the system with E, despite its crystalline nature, in the SNP-E it was not possible to detect any endothermic effect due to drug melting because of the low drug content and its molecular dispersion.

**Figure 3:**
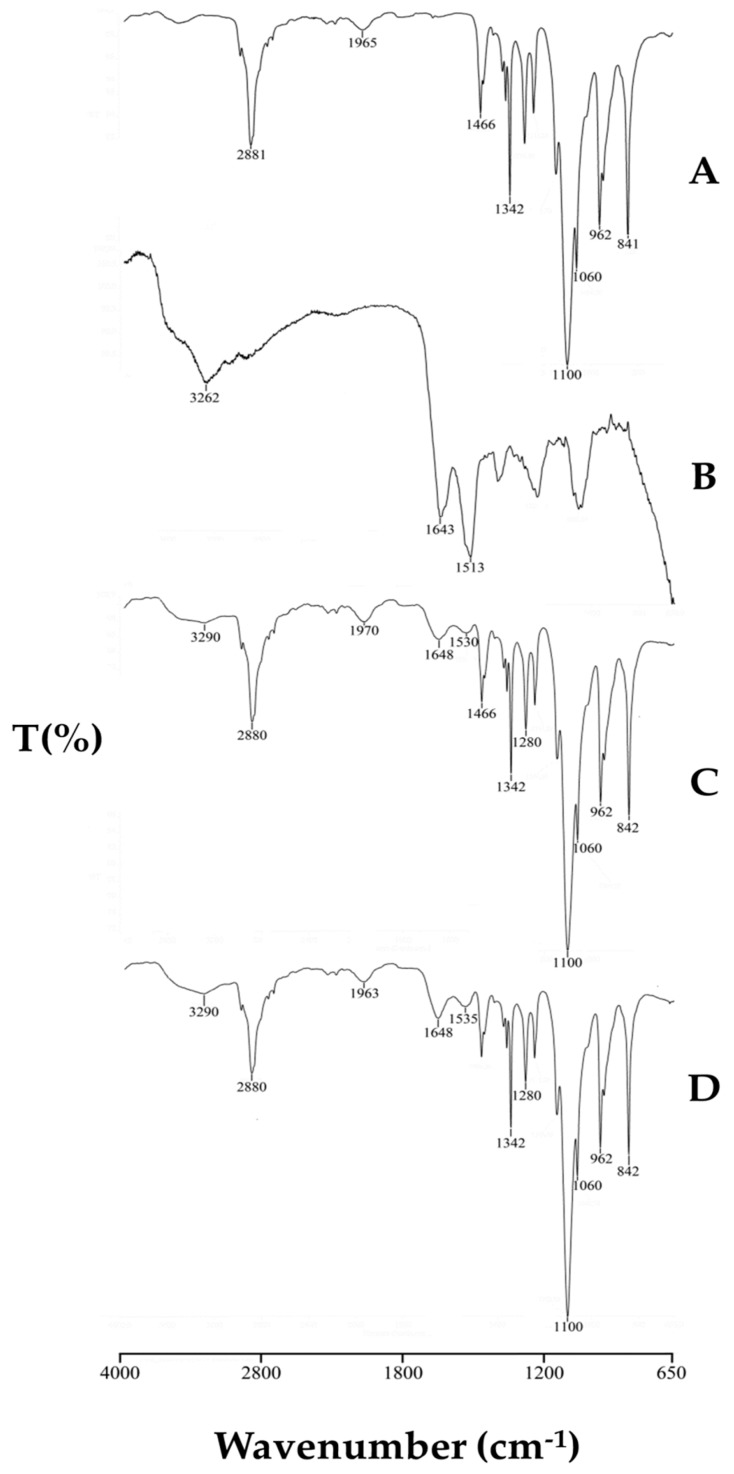
FT-IR spectra of Lutrol^®^ F127 (**A**), sericin (**B**), unloaded SNP (**C**) and loaded SNP-Q (**D**).

In Figure 3, FT-IR spectra of Lutrol^®^ F127, SS as well as unloaded and loaded SNPs are reported. The spectrum of Lutrol^®^ F127 shows a band at 2882 cm^−1^ due to C–H stretching vibration and a band at 1466 cm^−1^ due to C–H bending vibration. SS exhibited broadband peaked at 3262 cm^−1^ due to the stretching of the N–H bond of amides in concomitance with the absorption of the O–H groups, and the typical bands of C–O stretching at 1643 and N–H bending at 1513 cm^−1^ of amide I and II, respectively. In the FT-IR spectrum of unloaded SNPs, these bands are still visible, confirming the presence of the protein in the polymer system, but shifted to higher wavenumbers, 1648 and 1530 cm^−1^, respectively, as a consequence of silk-sericin poloxamer nanoparticles formulation causing SS transition into the β-sheet structure, as also highlighted by the thermal data. This spectrum is superimposable to that of the loaded SNPs. The characteristic signals of Q (not reported in the figure) in the region between 1700–1100 cm^−1^ were masked in the recorded spectrum of SNP-Q by the intense signals of SS. The same results are recorded also for the SNP-E. Instead, for the SNP-P system, the FT-IR spectrum is not reported because the amorphous character of the drug causes a broadening of the bands with a final bad resolution.

**Figure 4:**
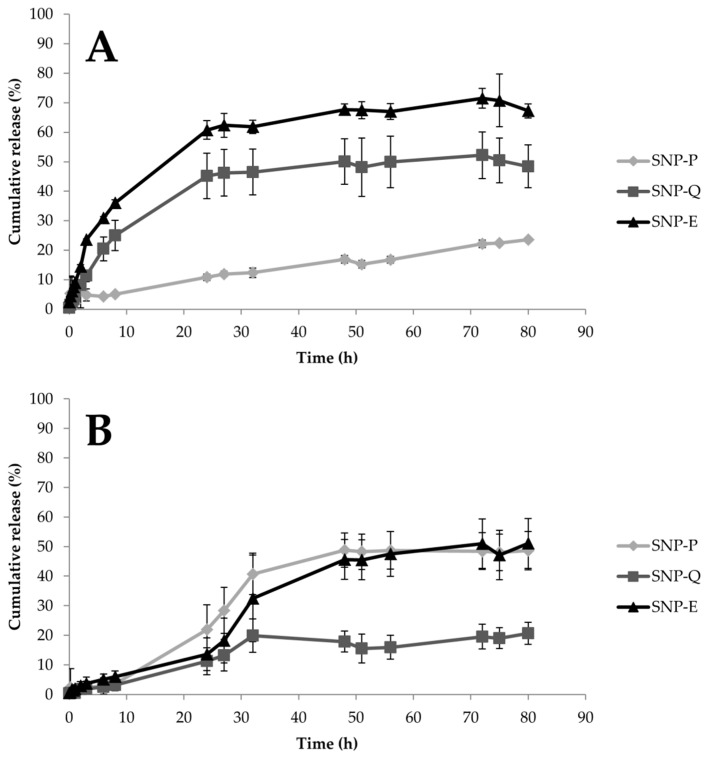
In vitro drug release profiles for SNPs in EtOH/water 50/50 *v*/*v* (**A**) or PBS (for SNP-P and SNP-E) and PBS + polysorbate 20 (for Q) (**B**). Data are reported as the cumulative drug release percentage (mean values ± standard deviation, *n* = 9) of at least three independent experiments for each batch.

Drug release from SNPs was investigated considering two dissolution media, and the data are reported in Figure 4 as a function of time. For both of the dissolution media, statistical analysis showed that formulation and time were statistically significant in influencing SNP performance (*p* < 0.05). In EtOH, SNP-E and SNP-Q showed a burst release; after 8 h, up to 36% and 25% of the whole loaded drug was released, respectively. At the same time, SNP-P released only 5% of the whole drug (Figure 4A). A plateau, corresponding to about 60%, 50% and 25% of the whole drug released, was observed after 48 h for SNP-E, SNP-Q and SNP-P, respectively. In PBS (or PBS + polysorbate 20 for Q), all the actives were released in a controlled manner. The hydrophilic actives were more easily released; after 48 h, the time at which a plateau was reached, SNP-P and SNP-E released 50% of the whole loaded drug, while SNP-Q released only 20% (Figure 4B). At the end of the drug release tests, the amount of actives not released from SNPs (or that remained in the membranes) was determined. The results confirmed the mass balance (Supplementary Materials).

Release profiles suggest a destructuration of the micellar structure of SNPs in EtOH, leading to an increased liberation rate of E and Q compared to what happens in PBS. On the other hand, the release of P is slower in EtOH compared to PBS. The chemical structures of actives, as well as the different solubility of SS in the release media, explain these different release profiles. Firstly, SS is less soluble in EtOH 50% *v*/*v* than in water, due to the lower polarity of the solvent. Also, P has complex chemical structure, in which catechins or flavanols are linked together by C–C bridges forming oligomers (from dimers to pentamers) or polymers (up to 60 units). P is bulkier than E and Q and it spreads slowly through SS (which in EtOH is less soluble). E and Q which have a lower molecular weight and steric hindrance than P, are released much faster. Moreover, it has to be considered that hydrophilic actives E and P reside in the hydrophilic external structure of SNPs (Figure 1A), and because they have a shorter path to follow, they are released more quickly. Q instead, being lipophilic, resides in the internal lipophilic core (Figure 1A) and has to spread through the hydrophilic layer made of SS and poloxamer. This latter aspect also explains the lower release of Q in PBS medium. Finally, the slow second-phase or lag-phase (observed for P and E in PBS) suggests a densely packed system with low porosity, and this effect could also be the result of pore closure, sericin–pluronic interactions, or drug sericin–pluronic drug interactions.

Drug release data were further processed by elaborating the kinetic model of the release systems. The goal of modeling the release process is to gain a deeper understanding of the release mechanisms of a specific material. Release models describe the release of the encapsulated molecules as a function of time and provide information about the exact mass transport mechanisms involved in the drug release. Higuchi, Peppas–Sahlin, Ritger–Peppas and zero-order are among the most employed models. Table 3 lists the results of in vitro release model fitting for SNP-P, SNP-Q and SNP-E. Different release behaviors were observed in the different dissolution media. Drug release from SNPs in PBS followed the Peppas–Shalin model, where both the Fickian contribution (first term of the equation) and the case-II relaxation contribution (second term of the equation) are considered [41]. Specifically, *k*_1_ is the constant related to the Fickian kinetics (diffusion constant); *k*_2_ is the constant related to case-II relaxation kinetics (erosion constant) and *m* is the diffusional exponent. As *k*_2_ > *k*_1_, and the *k*_1_ value is negative, it is indicative that case-II relaxation is predominant in the diffusion phenomenon in the release of the active substances from SNPs. This was also confirmed by the Ritger–Peppas model, for which proper fittings were calculated (R^2^ = 0.89 for P, 0.88 for E and 0.84 for Q): the *n* exponent was between 0.43 and 0.85, thus confirming the non-Fickian behavior. In EtOH, E and Q were released from SNPs in an almost-Fickian mode, while P was released by a Fickian diffusion. Indeed, according to Ritger–Peppas and Korsmeyer–Peppas models, *n* values for E and Q were 0.3654 and 0.417, respectively, while for P, the *n* value was 0.522. According to both equation models, *n* values lower than 0.5 are indicative of almost-Fickian diffusion, while an *n* value equal to 0.5 is indicative of pure Fickian diffusion. This behavior was also confirmed by the good fitting revealed for the Higuchi models, which describe the drug release as a diffusion process based on the Fick’s law, which is square root time dependent. Of note, *k* values were higher for E and Q with respect to P, thus confirming what was previously supposed regarding the lower diffusibility of P due to the higher steric hindrance of the molecule.

**Figure 5:**
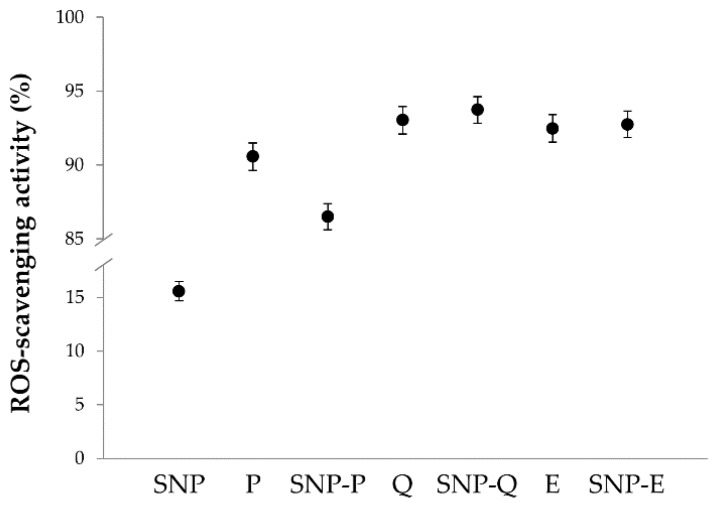
Results of average ROS-scavenging activity (%) as a function of SNP formulations (SNP, SNP-P, SNP-Q and SNP-E) and the equivalent amount of free actives (P, Q and E). Only data related to the highest concentration tested are reported (0.8 mg/mL for SNPs and the equivalent amount of free actives). Multifactor ANOVA, mean values ± LSD, *n* = 3.

The ROS-scavenging activity results are reported in Figure 5. The statistical analysis revealed that ROS-scavenging activity was significantly influenced by the sample (*p* < 0.001) but not by concentration (*p* > 0.05). All the active ingredients (P, Q and E) showed excellent antioxidant properties, with ROS-scavenging activity % values above 90%, even at the lowest concentration tested. For the unloaded SNPs, the average ROS-scavenging activity % was 15.60 ± 0.893. The encapsulation of Q and E into SNPs preserved their antioxidant activity as no significant differences were found between Q and SNP-Q or between E and SNP-E (*p* > 0.05). Conversely, for P the encapsulation into SNPs significantly reduced the ROS-scavenging activity % (*p* < 0.05). Overall, our data are in accordance with the literature, where antioxidant properties have been reported for P [42], Q [43], E [44] and sericin [11,18].

**Figure 6:**
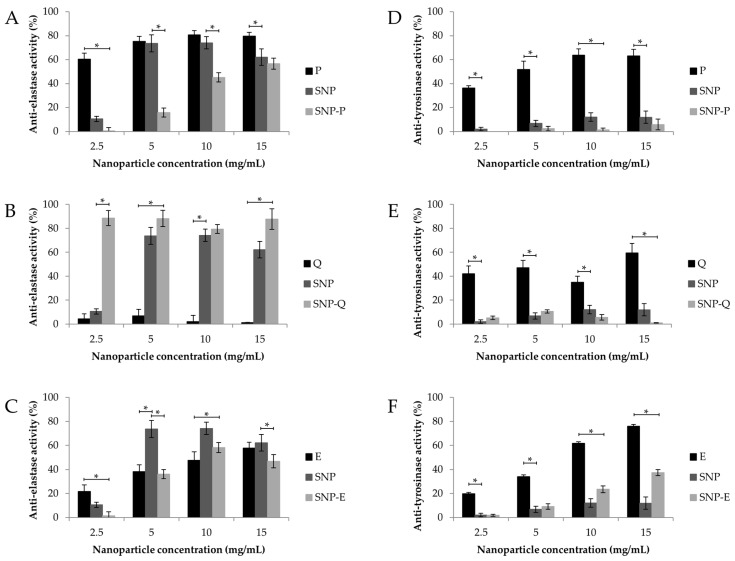
In vitro anti-elastase (**A**–**C**) and anti-tyrosinase (**D**–**F**) activity of SNP, SNP-P, SNP-Q and SNP-E, and an equivalent amount of free drug (P, Q and E). Data are reported as mean values ± standard deviation, *n* = 3. * *p* < 0.05.

In vitro biological activity of unloaded and loaded SNPs was subsequently assessed in terms of anti-elastase and anti-tyrosinase activity (Figure 6). Unloaded SNPs showed good intrinsic anti-elastase activity, which was higher than 70% starting from the 5 mg/mL concentration (Figure 6A–C). Therefore, the ability of SS to inhibit the elastase enzyme was also preserved when assembling the protein as a nanoparticle. Conversely, unloaded SNP showed low anti-tyrosinase activity at all of the concentrations tested (Figure 6D–F), despite the literature reports on the ability of SS to inhibit tyrosinase [11]. In this case, we supposed that the conformational changes in SS proteins, when assembled as a nanoparticle, hinder the interaction with the enzyme. Good anti-elastase activity was observed at all the concentrations tested, for both P and E (Figure 6A,C), thus confirming previous reports in the literature [45,46,47]. For SNP-P and SNP-E, the anti-elastase activity was lower compared to the equivalent amount of free drug (Figure 6A,C). A synergic anti-elastase effect was observed only for SNP-Q; after the encapsulation, the anti-elastase activity % of Q increased from 1.28 ± 0.168% to 87.7 ± 5.67% (at the highest concentration tested) (Figure 6B). In accordance with the literature, P and E showed a dose-dependent anti-tyrosinase activity. The anti-tyrosinase activity of both P and Q decreased after encapsulation into SNPs; only E, when encapsulated into SNPs, retained good inhibitory properties. The lower activity of loaded SNPs, with respect to the equivalent amount of free drugs, may be a result of (i) a lower amount of active (P, Q and E) that can interact and inhibit the enzyme, as it is not entirely released by SNPs after 24 h (see the release profiles reported in Figure 4); or (ii) the steric hindrance created by SNPs, which could hinder the link or interaction of the free compound/SS with the enzyme.

The enzymatic kinetics of anti-elastase and anti-tyrosinase activity were further elaborated with a model of Michaelis-Menten kinetics to extrapolate the Vmax and Km values, as reported in Table 4. For the Vmax of anti-elastase activity, no differences were observed among the samples and the negative control (*p* > 0.05), while the Km of unloaded SNPs was significantly higher than the negative control (*p* = 0.0107). Regarding the anti-tyrosinase activity, the statistical analysis revealed no differences in the Km and Vmax of all samples with respect to the negative control (*p* > 0.05). The calculation of Vmax and Km values provides more information about the inhibition mechanism. In particular, a distinction between enzyme inactivators and inhibitors can be made. Enzyme inactivators generally induce conformational changes, also mediated by the solvent molecules, in the tertiary and quaternary structure of the enzyme [48]. True inhibitors, instead, act according to a competitive, non-competitive, and mixed type (competitive/non-competitive) mechanism, modifying the kinetics of the enzyme, and thus Km and Vmax. According to the literature, flavonoids, such as P and Q, and catechins, such as E, act as elastase and tyrosinase inactivators by forming hydrophobic interactions with the enzymes, thus inducing a conformational change, or by chelating metals [45]. The SS mechanism of action in inhibiting elastase and tyrosinase enzymes has not yet been deeply investigated. It can be supposed that SS anti-elastase and anti-tyrosinase properties are the result of different mechanisms, which include the chelation of metals (such as copper and iron), the reducing ability of amino acids (e.g., serine, threonine, and aromatic amino acids) and the presence of secondary metabolites, such as flavonoids [49,50]. In this work, for both elastase and tyrosinase, all the samples were shown to be enzyme inactivators, with the exception of unloaded SNPs which increased Km and left Vmax unchanged, thus demonstrating they act as competitive inhibitors of elastase, directly binding to the active site of the enzyme.

**Figure 7:**
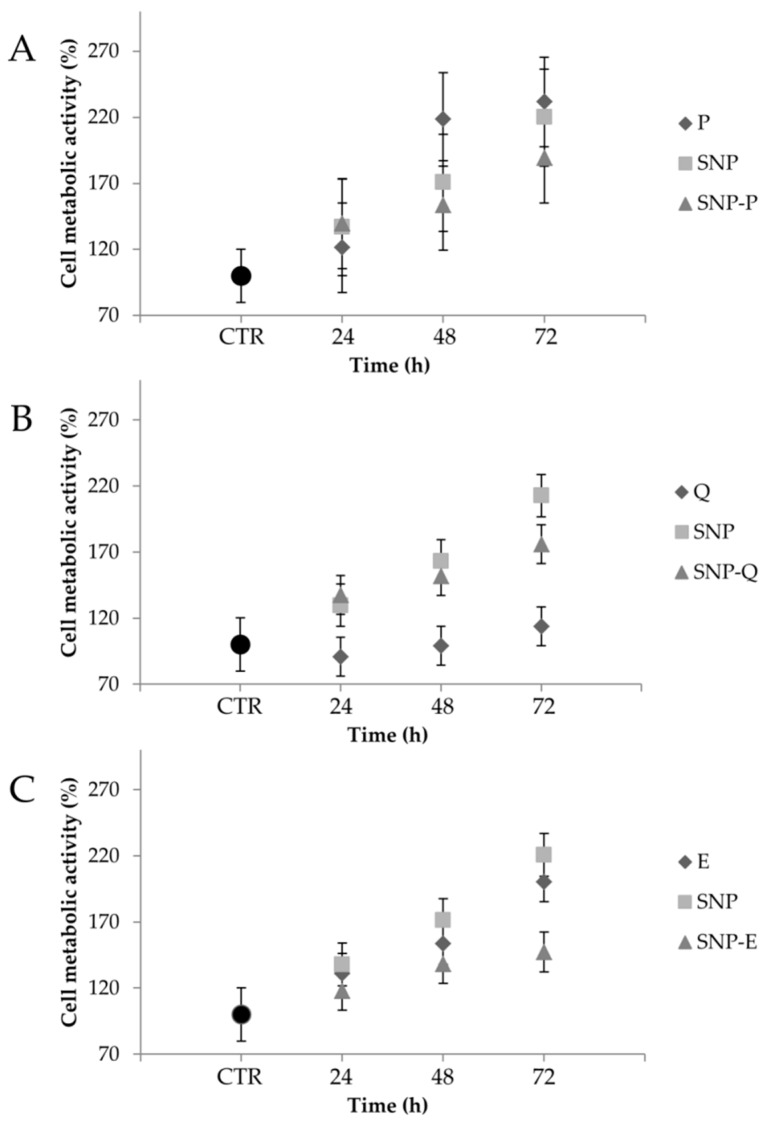
Cell metabolic activity % of mesenchymal stem/stromal cells (MSCs) treated with the highest dosage (0.8 mg/mL) of SNP, SNP-P (**A**), SNP-Q (**B**) and SNP-E (**C**), and the equivalent amount of free drug (P, Q and E). Untreated cells were considered as CTR (100% of metabolic activity). Multifactor ANOVA, mean values ± least significant difference (LSD), *n* = 3.

Figure 7 reports the percentage of MSC cell metabolic activity values after treatment, at the highest concentration (0.8 mg/mL), with unloaded and loaded SNPs and the equivalent amount of free actives. No cytotoxic effects were revealed. Statistical analysis revealed that sample concentration was not significant (*p* = 0.634); therefore, only the highest concentration tested (0.8 mg/mL) is reported in the figure. The metabolic activity of the untreated cells (CTR) was not modified over time (*p* > 0.05). Instead, after addition of all the samples, a time-dependent increase (*p* < 0.05) in the cell metabolic activity % was observed with respect to FBS-free medium (CTR). Statistical analysis revealed no differences among SNP, SNP-P and P (*p* > 0.05) at all considered times (Figure 7A). After 72 h, the cell metabolic activity of MSCs treated with E and unloaded SNPs was higher than SNP-E (*p* < 0.05). A significant increase in the cell metabolic activity was observed only for Q, at all the considered times, when encapsulated into SNPs (*p* < 0.05). The increased cell metabolic activity after treatment with SNPs can be related to the well-known mitogen effect of SS [18,51,52]. To the best of our knowledge, the proliferation ability of P has not been reported on MSCs, however, it has shown good regenerative properties on other cell lines [53]. Similar to the findings of Kim and colleagues, an inhibition in cell metabolic activity was observed when treating MSCs with Q [54]. This effect was avoided after encapsulation into SNPs. Finally, other authors have reported the ability of E in enhancing the cell proliferation and differentiation of adipose-derived MSCs [55]. Interestingly, the same authors reported that E enhanced MSC differentiation into endothelial progenitor cells, thus supporting the employment of such an active for tissue regeneration purposes.

**Figure 8:**
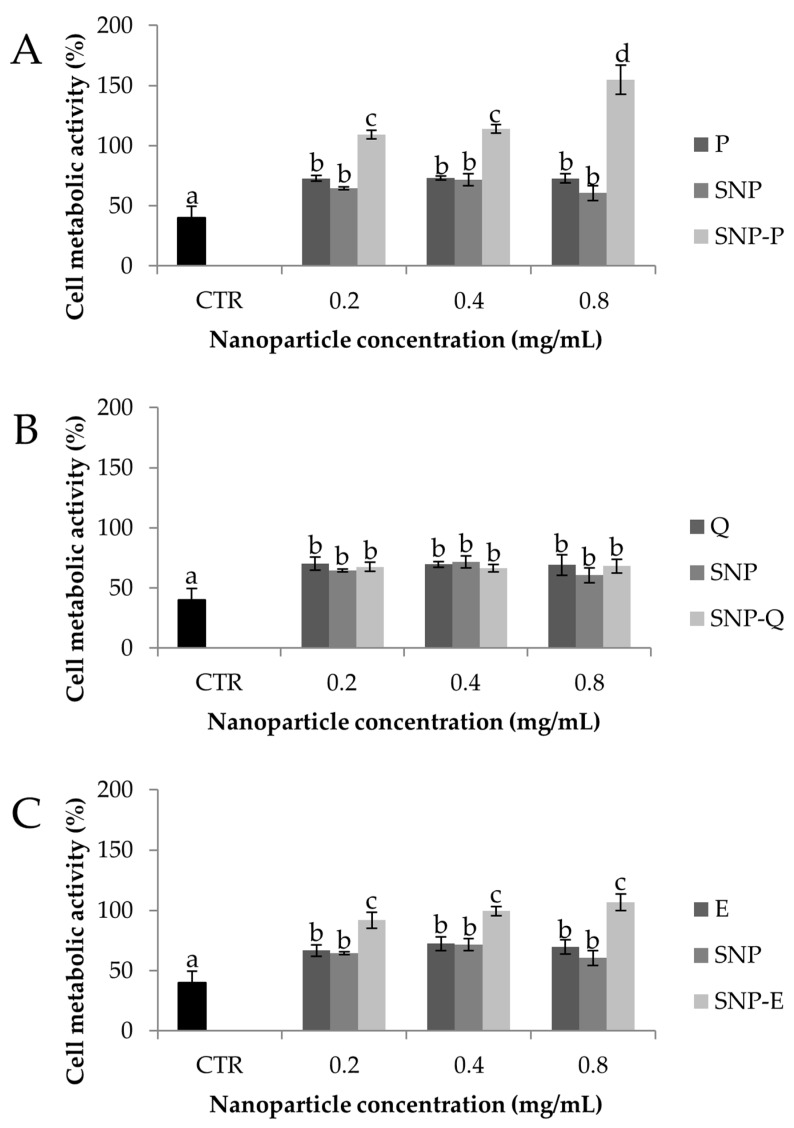
Cytoprotective properties of SNP-P (**A**), SNP-Q (**B**), SNP-E (**C**) compared to unloaded nanoparticles (SSNP) and the equivalent amount of free drugs on MSCs treated with H_2_O_2_ 1.5 mM for 24 h (CTR). Multifactor ANOVA, mean values ± least significant difference (LSD), *n* = 3. Different letters (a, b, c) indicate significant differences between the means (*p* < 0.05), whereas the same letter indicates no significant difference (*p* > 0.05).

Figure 8 compares the results of in vitro evaluation of SNP protective effect against oxidative damage of MSCs. Exposure to H_2_O_2_ 1.5 mM lowered the cell metabolic activity % significantly (*p* < 0.05), thus proving the suitability of the experimental conditions (both H_2_O_2_ concentration and contact time) in inducing oxidative damage. All the samples were shown to protect MSCs from oxidative stress as the cell metabolic activity % was significantly higher than CTR in their presence (*p* < 0.05). SNP-P highly protected MSCs against H_2_O_2_ damage (*p* < 0.05) in a dose-dependent manner, while no differences were found between P and SNP (*p* > 0.05). The same trend was observed for SNP-E, which protected cells much more significantly than E and SNP (*p* < 0.05). Instead, for Q, the encapsulation into SNPs did not increase the oxidative stress protection. Overall, the cytoprotective properties can be attributed to both SS and the active ingredients, for which the antioxidant properties are well-reported in the literature, as reported in the previous sections. Overall, the inclusion of the active in SNPs increased the cytoprotective properties, probably as a consequence of increased cell uptake, as previously demonstrated [28].

## 4. Conclusions

In this paper, SNPs were prepared by exploiting a self-assembly method with poloxamer for targeting naturally-derived flavonoids (P, Q and E) to MSCs. SNPs had a diameter less than 150 nm, rounded morphology, and were able to encapsulate both hydrophilic (P and E) and hydrophobic (Q) drugs, without significantly influencing particle size distribution or morphology. Physical-chemical characterization revealed that SNP integrity was preserved after drug encapsulation. A slow and controlled release profile was obtained from SNPs for all the actives in PBS, while in EtOH, a burst release was revealed for Q and E but not for P. Elaboration of drug release data by kinetic models revealed that P presents a lower diffusion index with respect to the other biomolecules, which is reasonable given their higher steric hindrance. For all the actives, in PBS drug release was mainly controlled by case-II relaxation, while in EtOH it was controlled by diffusion. All SNPs showed in vitro anti-oxidant, anti-elastase and anti-tyrosinase properties. The loading of P and E into SNPs preserved the in vitro biological activity, whereas for Q, the anti-elastase activity was strongly improved. All formulations promoted the metabolic activity of MSCs over 72 h, and protected cells against oxidative stress damage. Both findings can be related to the mitogen and antioxidant properties of both sericin and actives. Overall, the results reported in this paper support the employment of SNPs for targeting naturally-derived flavonoid to tissue resident MSCs for regenerative purposes.

## Figures and Tables

**Table 1:** Formulations considered for the study.

Formulation	Active Ingredient	Theoretical Drug Loading (%, *w*/*w*)
SNP	/	/
SNP-P	Proanthocyanidins (P)	3.2
SNP-Q	Quercetin (Q)	3.2
SNP-E	Epigallocatechin gallate (E)	3.2

**Table 2:** SNP formulations and composition details. The process yield (%), drug loading (%) and encapsulation efficiency (%) are reported as mean values ± standard deviation, *n* = 3, of at least three independent experiments for each batch. Different letters (^a^, ^b^, ^c^) among the same column correspond to significant differences between groups (*p* < 0.05), while the same letter indicates no significant differences (*p* < 0.05).

Nanoparticle Formulation	Process Yield (%)	Drug Loading (% *w*/*w*)	Encapsulation Efficiency (%)
SNP	62.6 ± 5.68 ^a^	-	-
SNP-P	63.8 ± 4.25 ^a^	2.6 ± 0.37 ^a^	82.2 ± 11.58 ^a^
SNP-Q	60.9 ± 0.46 ^a^	0.7 ± 0.21 ^b^	20.5 ± 6.59 ^b^
SNP-E	63.7 ± 1.79 ^a^	1.3 ± 0.17 ^c^	41.5 ± 5.25 ^c^

**Table 3:** Results of in vitro release model fitting for SNP-P, SNP-Q and SNP-E. Kinetic elaborations were performed on release data obtained from at least three independent experiments for each batch. ~ indicates that the analysis performed was “ambiguous”; therefore, the fit does not nail down the values of all the parameters, and 95% confidence bounds cannot be reported. These latter data were not considered in the interpretation of results.

Model	Equation	Sample	Dissolution Medium	Coefficients (95% Confidence Bounds)	Sum of Squares	R^2^	Degrees of Freedom	SE
**Higuchi**	*F*(*t*) = *k* × *t*^0.5^	SNP-E	PBS	*k* = 5.516 (5.261, 5.771)	11,734	0.8421	161	0.1290
			EtOH	*k* = 9.265 (8.821, 9.709)	3787	0.9051	53	0.2213
		SNP-Q	PBS	*k* = 2.319 (2.137, 2.501)	635.8	0.8396	53	0.09066
			EtOH	*k* = 6.895 (6.620, 7.170)	13,653	0.8378	161	0.1392
		SNP-P	PBS	*k* = 5.801 (5.564, 6.039)	10,214	0.8694	161	0.1204
			EtOH	*k* = 2.432 (2.285, 2.579)	416.5	0.868	53	0.07338
**Higuchi** **(eq 2.12 from [31])**	*F*(*t*) = 100 × (1 − C × exp^(−*k*×*t*)^)	SNP-E	PBS	C = 0.9979 (0.9819, 1.014) *k* = 0.009999 (0.009381, 0.01063)	7962	0.8928	160	C 0.008122 *k* 0.0003151
			EtOH	C = 0.8826 (0.8430, 0.9226) *k* = 0.02064 (0.01780, 0.02389)	4440	0.8888	52	C 0.01936 *k* 0.00136
		SNP-Q	PBS	C = 0.9837 (0.9687, 0.9987) *k* = 0.003084 (0.002645, 0.003528)	786.6	0.8016	52	C 0.007455 *k* 0.0002181
			EtOH	C = 0.9222 (0.8972, 0.9472) *k* = 0.0118 (0.01064, 0.01300)	18,407	0.7813	160	C 0.01246 *k* 0.0005668
		SNP-P	PBS	C = 0.9947 (0.9785, 1.011) *k* = 0.01071 (0.01005, 0.01138)	8025	0.8974	160	C 0.008183 *k* 0.0003287
			EtOH	*C* = 0.9658 (0.9562, 0.9755) *k* = 0.002922 (0.002637, 0.003208)	327.5	0.8962	52	C 0.004806 *k* 0.0001423
**Peppas–Sahlin**	*F*(*t*) = *k*_1_ × *t^m^* + *k*_2_ × *t*^(2×*m*)^	SNP-E	PBS	*k*_1_ = −3.034 (−8.640, 1.090) *k*_2_ = 3.714 (1.074, 7.992) *m* = 0.3323 (0.2638, 0.4380)	8357	0.8875	159	*k*_1_ 2.849 *k*_2_ 2.02 *m* 0.04814
			EtOH	*k*_1_~ *k*_2_~ *m*~	1090	0.9727	51	*k*_1_~ *k*_2_~ *m*~
		SNP-Q	PBS	*k*_1_ = −7.5 (−27.08 to 0.4361) *k*_2_ = 8.15 (−0.06646 to 27.32) *m* = 0.1724 (0.08940, 0.3108)	577.1	0.8544	51	*k*_1_ 6.388 *k*_2_ 5.974 *m* 0.05588
			EtOH	*k*_1_~ *k*_2_~ *m*~	10,742	0.8724	159	*k*_1_~ *k*_2_~ *m*~
		SNP-P	PBS	*k*_1_ = −11.42 (−20.48, −4.672) *k*_2_ = 11.6 (6.151, 19.67) *m* = 0.2289 (0.1850, 0.2822)	7805	0.9002	159	*k*_1_ 4.707 *k*_2_ 4.058 *m* 0.02951
			EtOH	*k*_1_~ *k*_2_~ *m*~	388.6	0.8768	51	*k*_1_~ *k*_2_~ *m*~
**Ritger–Peppas**	*F*(*t*) = *k* × *t^n^*	SNP-E	PBS	*k* = 1.783 (1.226, 2.496) *n* = 0.7854 (0.7028, 0.8766)	8458	0.8861	160	*k* 0.3431 *n* 0.04708
			EtOH	*k* = 15.63 (13.45, 17.91) *n* = 0.3654 (0.3300, 0.4039)	2137	0.9465	52	*k* 1.207 *n* 0.02005
		SNP-Q	PBS	*k* = 1.744 (1.011, 2.692) *n* = 0.5727 (0.4632, 0.7069)	616.2	0.8446	52	*k* 0.4702 *n* 0.06732
			EtOH	*k* = 9.531 (7.955, 11.21) *n* = 0.4169 (0.3751, 0.4626)	12,705	0.849	160	*k* 0.9111 *n* 0.0245
		SNP-P	PBS	*k* = 2.983 (2.248, 3.852) *n* = 0.6691 (0.6057, 0.7385)	8480	0.8916	160	*k* 0.4643 *n* 0.03845
			EtOH	*k* = 2.232 (1.346, 3.297) *n* = 0.522 (0.4223, 0.6480)	415.2	0.8684	52	*k* 0.4251 *n* 0.04789
**Zero order**	*F*(*t*) = *k* × *t*	SNP-E	PBS	*k* = 0.7373 (0.7070, 0.7676)	9473	0.8725	161	0.01536
			EtOH	*k* = 1.156 (1.027, 1.284)	18,129	0.5458	53	0.06416
		SNP-Q	PBS	*k* = 0.3012 (0.2718, 0.3306)	948.1	0.7608	53	0.01467
			EtOH	*k* = 0.8692 (0.8122, 0.9263)	33,494	0.602	161	0.02889
		SNP-P	PBS	*k* = 0.7643 (0.7302, 0.7984)	11,981	0.8468	161	0.01728
			EtOH	*k* = 0.3172 (0.2920, 0.3423)	694.3	0.7799	53	0.01256
**Korsmeyer–Peppas**	*F*(*t*) = *k_KP_* × *t^n^* × Q_0_	SNP-E	PBS	*k_KP_* = 1.783 (1.226, 2.496) *n* = 0.7854 (0.7028, 0.8766)	8458	0.8861	160	*k_KP_* 0.3431 *n* 0.04708
			EtOH	*k_KP_* = 15.63 (13.45, 17.91) *n* = 0.3654 (0.3300, 0.4039)	2137	0.9465	52	*k_KP_* 1.207 *n* 0.02005
		SNP-Q	PBS	*k_KP_* = 1.744 (1.011, 2.692) *n* = 0.5727 (0.4632, 0.7069)	616.2	0.8446	52	*k_KP_* 0.4702 *n* 0.06732
			EtOH	*k_KP_* = 9.531 (7.955, 11.21) *n* = 0.4169 (0.3751, 0.4626)	12,705	0.849	160	*k_KP_* 0.9111 *n* 0.0245
		SNP-P	PBS	*k_KP_* = 2.983 (2.248, 3.852) *n* = 0.6691 (0.6057, 0.7385)	8480	0.8916	160	*k_KP_* 0.4643 *n* 0.03845
			EtOH	*k_KP_* = 2.232 (1.346, 3.297) *n* = 0.522 (0.4223, 0.6480)	415.2	0.8684	52	*k_KP_* 0.4251 *n* 0.04789

**Table 4:** Vmax and Km values for each sample analyzed. Multifactor ANOVA, mean values ± standard error, *n* = 9. Different letters (^a^, ^b^, ^c^) indicate significant differences between the means (*p* < 0.05), whereas the same letter indicates no significant difference (*p* > 0.05).

	Anti-Elastase Activity				Anti-Tyrosinase Activity			
Sample	Km		Vmax		Km		Vmax	
	Mean	SE	Mean	SE	Mean	SE	Mean	SE
SNP	233.65 ^a^	51.147 ^a^	2.94 ^a^	0.982	66.91 ^a,b^	39.928	2.71 ^a,b,c^	0.940
SNP-P	37.30 ^b,c^	36.166 ^a,b^	1.09 ^a,b^	0.694	78.37 ^a,b^	46.105	4.11 ^b,c^	1.086
SNP-Q	9.74 ^b,c^	36.166 ^a,b^	0.80 ^a,b^	0.694	91.48 ^a,b^	39.928	4.67 ^c^	0.940
SNP-E	109.145 ^a,b^	36.166 ^a,b^	2.00 ^a,b^	0.694	183.37 ^b^	46.105	4.68 ^b,c^	1.086
P	47.16 ^b,c^	36.166 ^b^	0.46 ^b^	0.694	14.47 ^a^	39.928	0.43 ^a^	0.941
Q	10.02 ^b,c^	36.166 ^a,b^	0.78 ^a,b^	0.694	81.79 ^a,b^	46.105	1.55 ^a,b^	1.085
E	41.37 ^b,c^	21.809 ^b^	0.51 ^b^	0.419	226.5 ^b^	79.857	6.69 ^c^	1.881
CTR -	10.50 ^c^	27.339 ^a,b^	1.83 ^a,b^	0.525	42.09	3.018	2.12 ^a,b^	0.711

## Supplementary Materials

The Supplementary Materials are available online at https://www.mdpi.com/1999-4923/12/4/381/s1. Isolation and expansion of human adipose-derived mesenchymal stromal cells; Experiment to confirm drug loading and drug release; Figure S1. Drug loading (% *w*/*w*) of different batches (1, 2 and 3) for SNPs loaded with P, Q and E. Multifactor ANOVA, mean values ± least significant difference (LSD), *n* = 3.

## References

[1] Fu Y., Karbaat L., Wu L., Leijten J., Both S.K., Karperien M. (2017). Trophic Effects of Mesenchymal Stem Cells in Tissue Regeneration. Tissue Eng. Part B Rev..

[2] Li Y., Wu Q., Wang Y., Li L., Bu H., Bao J. (2017). Senescence of mesenchymal stem cells. Int. J. Mol. Med..

[3] Bari E., Ferrarotti I., Torre M.L., Corsico A.G., Perteghella S. (2019). Mesenchymal stem/stromal cell secretome for lung regeneration: The long way through “pharmaceuticalization” for the best formulation. J. Control. Release.

[4] Zhang D., Chen Y., Xu X., Xiang H., Shi Y., Gao Y., Wang X., Jiang X., Li N., Pan J. (2020). Autophagy inhibits the mesenchymal stem cell aging induced by d-galactose through ROS/JNK/p38 signalling. Clin. Exp. Pharmacol. Physiol..

[5] Denu R.A., Hematti P. (2016). Effects of Oxidative Stress on Mesenchymal Stem Cell Biology. Oxidative Med. Cell. Longev..

[6] Alexander V.A.D., Radhakrishnan A., Subramani P. (2016). Overviews of Biological Importance of Quercetin: A Bioactive Flavonoid. Pharmacogn. Rev.

[7] Rauf A., Imran M., Abu-Izneid T., Iahfisham Ul H., Patel S., Pan X.D., Naz S., Silva A.S., Saeed F., Suleria H.A.R. (2019). Proanthocyanidins: A comprehensive review. Biomed. Pharmacother..

[8] Bartosikova L., Necas J. (2018). Epigallocatechin gallate: A review. Veterinarni Med..

[9] McCarty S.M., Percival S.L. (2013). Proteases and Delayed Wound Healing. Adv. Wound Care.

[10] Wang Y.-J., Zhang Y.-Q. (2011). Three-layered sericins around the silk fibroin fiber from Bombyx mori cocoon and their amino acid composition. Silk Inherit. Innov. Mod. Silk Road.

[11] Chlapanidas T., Farago S., Lucconi G., Perteghella S., Galuzzi M., Mantelli M., Avanzini M.A., Tosca M.C., Marazzi M., Vigo D. (2013). Sericins exhibit ROS-scavenging, anti-tyrosinase, anti-elastase, and in vitro immunomodulatory activities. Int. J. Biol. Macromol..

[12] Lamboni L., Gauthier M., Yang G., Wang Q. (2015). Silk sericin: A versatile material for tissue engineering and drug delivery. Biotechnol Adv..

[13] Bari E., Arciola C.R., Vigani B., Crivelli B., Moro P., Marrubini G., Sorrenti M., Catenacci L., Bruni G., Chlapanidas T. (2017). In Vitro Effectiveness of Microspheres Based on Silk Sericin and Chlorella vulgaris or Arthrospira platensis for Wound Healing Applications. Materials.

[14] Crivelli B., Perteghella S., Bari E., Sorrenti M., Tripodo G., Chlapanidas T., Torre M.L. (2018). Silk nanoparticles: From inert supports to bioactive natural carriers for drug delivery. Soft Matter.

[15] Bari E., Perteghella S., Farago S., Torre M.L. (2018). Association of silk sericin and platelet lysate: Premises for the formulation of wound healing active medications. Int. J. Biol. Macromol..

[16] Aramwit P., Siritientong T., Srichana T. (2012). Potential applications of silk sericin, a natural protein from textile industry by-products. Waste Manag. Res..

[17] Nardini M., Perteghella S., Mastracci L., Grillo F., Marrubini G., Bari E., Formica M., Gentili C., Cancedda R., Torre M.L. (2020). Growth Factors Delivery System for Skin Regeneration: An Advanced Wound Dressing. Pharmaceutics.

[18] Bari E., Perteghella S., Marrubini G., Sorrenti M., Catenacci L., Tripodo G., Mastrogiacomo M., Mandracchia D., Trapani A., Farago S. (2018). In vitro efficacy of silk sericin microparticles and platelet lysate for intervertebral disk regeneration. Int. J. Biol. Macromol..

[19] Aramwit P., Yamdech R., Ampawong S. (2016). Controlled Release of Chitosan and Sericin from the Microspheres-Embedded Wound Dressing for the Prolonged Anti-microbial and Wound Healing Efficacy. AAPS J..

[20] Suktham K., Koobkokkruad T., Wutikhun T., Surassmo S. (2018). Efficiency of resveratrol-loaded sericin nanoparticles: Promising bionanocarriers for drug delivery. Int. J. Pharm..

[21] Mandal B.B., Kundu S.C. (2009). Self-assembled silk sericin/poloxamer nanoparticles as nanocarriers of hydrophobic and hydrophilic drugs for targeted delivery. Nanotechnology.

[22] Cho K.Y., Moon J.Y., Lee Y.W., Lee K.G., Yeo J.H., Kweon H.Y., Kim K.H., Cho C.S. (2003). Preparation of self-assembled silk sericin nanoparticles. Int. J. Biol. Macromol..

[23] Parisi O.I., Fiorillo M., Scrivano L., Sinicropi M.S., Dolce V., Iacopetta D., Puoci F., Cappello A.R. (2015). Sericin/Poly(ethylcyanoacrylate) Nanospheres by Interfacial Polymerization for Enhanced Bioefficacy of Fenofibrate: In Vitro and In Vivo Studies. Biomacromolecules.

[24] Huang L., Tao K., Liu J., Qi C., Xu L., Chang P., Gao J., Shuai X., Wang G., Wang Z. (2016). Design and Fabrication of Multifunctional Sericin Nanoparticles for Tumor Targeting and pH-Responsive Subcellular Delivery of Cancer Chemotherapy Drugs. ACS Appl. Mater. Interfaces.

[25] Kanoujia J., Singh M., Singh P., Saraf S.A. (2016). Novel genipin crosslinked atorvastatin loaded sericin nanoparticles for their enhanced antihyperlipidemic activity. Mater. Sci. Eng. C Mater. Biol. Appl..

[26] Hazeri N., Tavanai H., Moradi A.R. (2012). Production and properties of electrosprayed sericin nanopowder. Sci. Technol. Adv. Mater..

[27] Perteghella S., Crivelli B., Catenacci L., Sorrenti M., Bruni G., Necchi V., Vigani B., Sorlini M., Torre M.L., Chlapanidas T. (2017). Stem cell-extracellular vesicles as drug delivery systems: New frontiers for silk/curcumin nanoparticles. Int. J. Pharm..

[28] Guo W., Deng L., Yu J., Chen Z., Woo Y., Liu H., Li T., Lin T., Chen H., Zhao M. (2018). Sericin nanomicelles with enhanced cellular uptake and pH-triggered release of doxorubicin reverse cancer drug resistance. Drug Deliv..

[29] Couvreur P. (2013). Nanoparticles in drug delivery: Past, present and future. Adv. Drug Deliv. Rev..

[30] Crivelli B., Bari E., Perteghella S., Catenacci L., Sorrenti M., Mocchi M., Farago S., Tripodo G., Prina-Mello A., Torre M.L. (2019). Silk fibroin nanoparticles for celecoxib and curcumin delivery: ROS-scavenging and anti-inflammatory activities in an in vitro model of osteoarthritis. Eur. J. Pharm. Biopharm..

[31] Caccavo D. (2019). An overview on the mathematical modeling of hydrogels’ behavior for drug delivery systems. Int. J. Pharm..

[32] Bari E., Perteghella S., Di Silvestre D., Sorlini M., Catenacci L., Sorrenti M., Marrubini G., Rossi R., Tripodo G., Mauri P. (2018). Pilot Production of Mesenchymal Stem/Stromal Freeze-Dried Secretome for Cell-Free Regenerative Nanomedicine: A Validated GMP-Compliant Process. Cells.

[33] della Cuna F.S.R., Calevo J., Bari E., Giovannini A., Boselli C., Tava A. (2019). Characterization and Antioxidant Activity of Essential Oil of Four Sympatric Orchid Species. Molecules.

[34] Bari E., Ferrarotti I., Di Silvestre D., Grisoli P., Barzon V., Balderacchi A., Torre M.L., Rossi R., Mauri P., Corsico A.G. (2019). Adipose Mesenchymal Extracellular Vesicles as α-1-Antitrypsin Physiological Delivery Systems for Lung Regeneration. Cells.

[35] Dominici M., Le Blanc K., Mueller I., Slaper-Cortenbach I., Marini F.C., Krause D.S., Deans R.J., Keating A., Prockop D.J., Horwitz E.M. (2006). Minimal criteria for defining multipotent mesenchymal stromal cells. The International Society for Cellular Therapy position statement. Cytotherapy.

[36] Hanafy N.A.N., El-Kemary M., Leporatti S. (2018). Micelles Structure Development as a Strategy to Improve Smart Cancer Therapy. Cancers.

[37] Khonkarn R., Mankhetkorn S., Hennink W.E., Okonogi S. (2011). PEG-OCL micelles for quercetin solubilization and inhibition of cancer cell growth. Eur. J. Pharm. Biopharm..

[38] Li Y., Yang L. (2015). Driving forces for drug loading in drug carriers. J. Microencapsul..

[39] Sunoqrot S., Alsadi A., Tarawneh O., Hamed R. (2017). Polymer type and molecular weight dictate the encapsulation efficiency and release of Quercetin from polymeric micelles. Colloid Polym. Sci..

[40] Catenacci L., Sorrenti M., Bruni G., Bonferoni C.M., Sandri G., Bettinetti G. (2013). Characterization of silver sulfadiazine-loaded solid lipid nanoparticles by thermal analysis. J. Therm. Anal. Calorim..

[41] Peppas N.A., Sahlin J.J. (1989). A simple equation for the description of solute release. 3. Coupling of diffusion and relaxation. Int. J. Pharm..

[42] Park Y.S., Jeon M.H., Hwang H.J., Park M.R., Lee S.-H., Kim S.G., Kim M. (2011). Antioxidant activity and analysis of proanthocyanidins from pine (*Pinus densiflora*) needles. Nutr. Res. Pract..

[43] Hatahet T., Morille M., Shamseddin A., Aubert-Pouessel A., Devoisselle J.M., Begu S. (2017). Dermal quercetin lipid nanocapsules: Influence of the formulation on antioxidant activity and cellular protection against hydrogen peroxide. Int. J. Pharm..

[44] Legeay S., Rodier M., Fillon L., Faure S., Clere N. (2015). Epigallocatechin Gallate: A Review of Its Beneficial Properties to Prevent Metabolic Syndrome. Nutrients.

[45] Hong Y.-H., Jung E.Y., Noh D.O., Suh H.J. (2014). Physiological effects of formulation containing tannase-converted green tea extract on skin care: Physical stability, collagenase, elastase, and tyrosinase activities. Integr. Med. Res..

[46] Pientaweeratch S., Panapisal V., Tansirikongkol A. (2016). Antioxidant, anti-collagenase and anti-elastase activities of Phyllanthus emblica, Manilkara zapota and silymarin: An in vitro comparative study for anti-aging applications. Pharm. Biol..

[47] Bos M.A., Vennat B., Meunier M.T., Pouget M.P., Pourrat A., Fialip J. (1996). Procyanidins from tormentil: Antioxidant properties towards lipoperoxidation and anti-elastase activity. Biol. Pharm. Bull..

[48] Zolghadri S., Bahrami A., Khan M.T.H., Munoz-Munoz J., Garcia-Molina F., Garcia-Canovas F., Saboury A.A. (2019). A comprehensive review on tyrosinase inhibitors. J. Enzyme Inhib. Med. Chem..

[49] Kato N., Sato S., Yamanaka A., Yamada H., Fuwa N., Nomura M. (1998). Silk protein, sericin, inhibits lipid peroxidation and tyrosinase activity. Biosci. Biotechnol. Biochem..

[50] Kubo I., Kinst-Hori I. (1999). Flavonols from saffron flower: Tyrosinase inhibitory activity and inhibition mechanism. J. Agric. Food Chem..

[51] Cao T.-T., Zhang Y.-Q. (2016). Processing and characterization of silk sericin from Bombyx mori and its application in biomaterials and biomedicines. Mater. Sci. Eng. C Mater. Biol. Appl..

[52] Sapru S., Das S., Mandal M., Ghosh A.K., Kundu S.C. (2019). Nonmulberry silk protein sericin blend hydrogels for skin tissue regeneration—In vitro and in vivo. Int. J. Biol. Macromol..

[53] Kulakowski D., Leme-Kraus A.A., Nama J.-W., McAlpine J., Chen S.-N., Pauli G.F., Ravindran S., Bedran-Russo A.K. (2017). Oligomeric proanthocyanidins released from dentin induce regenerative dental pulp cell response. Acta Biomater..

[54] Kim Y.J., Bae Y.C., Suh K.T., Jung J.S. (2006). Quercetin, a flavonoid, inhibits proliferation and increases osteogenic differentiation in human adipose stromal cells. Biochem. Pharm..

[55] Widowati W., Tan Sardjono C., Wijaya C., Laksmitawati D.R., Sandra F. (2012). Extract of *Curcuma longa* L. and (-)-Epigallo Catechin-3-Gallate Enhanced Proliferation of Adipose Tissue–derived Mesenchymal Stem Cells (AD-MSCs) and Differentiation of AD-MSCs into Endothelial Progenitor Cells. J. US China Med. Sci..

