# Supplementary material for: Polyphenols-Loaded Sericin Self-Assembling Nanoparticles: A Slow-Release for Regeneration by Tissue-Resident Mesenchymal Stem/Stromal Cells
Source: Pharmaceutics. 2020 Apr 21;12(4):381. doi: 10.3390/pharmaceutics12040381 (PMC7238282; doi:10.3390/pharmaceutics12040381)
Supplement: Supplementary file 1 [file pharmaceutics-12-00381-s001.pdf]

pharmaceutics

MDPI

Supplementary Materials: Polyphenols-Loaded Sericin Self-Assembling Nanoparticles: A Slow-Release for Regeneration by Tissue-Resident Mesenchymal Stem/Stromal Cells

Giulia Orlandi, Elia Bari, Laura Catenacci, Milena Sorrenti, Lorena Segale, Silvio Faragò, Marzio Sorlini, Carla Renata Arciola, Maria Luisa Torre and Sara Perteghella

Isolation and Expansion of Human Adipose-Derived Mesenchymal Stromal Cells

Adipose tissues were obtained from three patients (mean age was 47 ± 4) undergoing abdominoplasty surgery after informed consent (ASST Grande Ospedale Metropolitano Niguarda, Milan). Clinical sheets of donors (identity, gender, age, tissue processing, sampling site, the day of collection and anamnesis) were filed by the surgery-responsible structure. Donors with septicemia or extensive infections, syphilis, type B and C hepatitis, HIV, Creutzfeld-Jacobs disease, viral or unknown neurological diseases, human GH treatment, and malignant tumours were excluded from the trial. Tissue samples were repeatedly washed with Phosphate Buffer Saline (PBS, pH= 7.14) without Ca^{2+} and Mg^{2+} and mechanically minced by surgical scissors. Tissue digestion was performed with 0.075% *(w/v)* type II collagenase solubilized in PBS with Ca^{2+} and Mg^{2+} plus 1% penicillin/streptomycin and 1% (*w/v*) amphotericin B at 37 °C. After 1 hour, Dulbecco's modified Eagle's Medium (DMEM) and Ham's medium F12 (DMEM/F12, ratio 1:1) supplemented by 10% (*v/v*) fetal bovine serum (FBS) was added to cell suspension; the digested tissue was filtered on 70 µm cell strainer (Greiner Bio-One, Milan, Italy) and centrifuged at 600 g for 5 minutes [1,2]. Recovered stromal vascular fraction was cultured in monolayer conditions (100,000 cells/cm^2) in DMEM F12, 10% (*v/v*) FBS, 1% (*v/v*) penicillin/streptomycin and 1% (*v/v*) amphotericin B. Once MSCs reached sub-confluence, they were treated with 0.05% (*v/v*) trypsin-EDTA and seeded onto flasks (10,000 cells/cm^2) at 37°C and 5% CO_2 and cultured until P6 in DMEM F12, 10% (*v/v*) FBS, 1% (*v/v*) penicillin/streptomycin and 1% (*v/v*) amphotericin B. All MSCs were tested to assure all the requirements needed for clinical use in terms of identity (according to the International Society for Cellular Therapy), sterility (according to Eu. Ph. 9.0, 2.6.27), tumorigenesis and karyotype.

Supplementary Figure

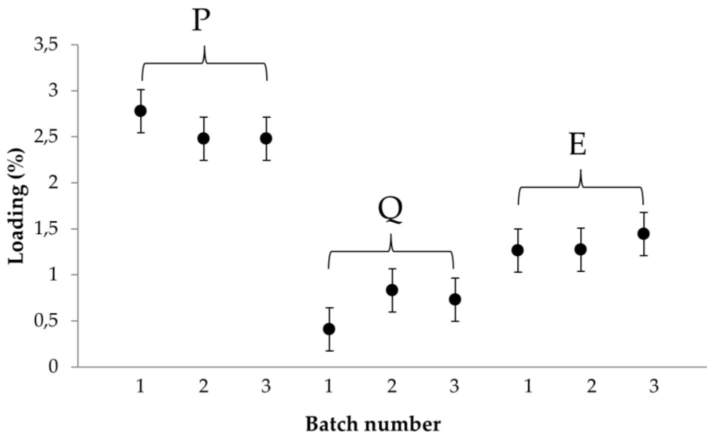

Figure S1. Drug loading (% *w/w*) of different batches (1, 2 and 3) for SNPs loaded with P, Q and E. Multifactor ANOVA, mean values ± least significant difference (LSD), *n* = 3.

Experiment to Confirm Drug Loading and Drug Release

An additional experiment was performed to confirm the drug loading and drug release data. Drug release experiments were repeated for each formulations in duplicate following the procedures reported in section 2.2.7. Briefly, for each batch 200 mg of SNPs were suspended in 5 ml of dissolution media and put into a dialysis membrane (3.5 kDa MWCO, Thermo Fisher Scientific, Milan, Italy). Each dialysis tube was incubated in 50 mL of dissolution media and maintained under mild magnetic stirring at 37 °C. After 80 h, the amount of released drug was determined by a spectrophotometric method (UV/VIS Spectrometer Lambda20, PerkinElmer, Wellesley, MA, USA) analyzing the release media at 279, 275 and 373 nm for P, E and Q, respectively. The drug concentration was extrapolated from a calibration curve previously prepared (P 5–80 μg/mL, R^2 = 0.99; Q 0.5–15 μg/mL, R^2 = 0.99; E 5–30 μg/mL, R^2 = 0.98). The cumulative amount of released drug was calculated in percentage using the following equation:

$$\text{Cumulative amount of drug released (\%)} = C_i/C_0 \times 100 \quad \text{(S1)}$$

where C_i was the amount of the drug released at 80 hours and C_0 was the loaded drug amount.

Then, SNPs inside the dialysis bag were recovered and bring to a final volume of 50 mL by using the dissolving media reported in the section 2.2.3. In detail, SNP-P and SNP-E were dissolved in deionized water plus HCl (0.1%, *v*/*v*), while SNP-Q were dissolved in ethanol 96% (*v*/*v*) maintaining mild magnetic stirring in the dark. The dialysis membranes were repeatedly washed with the abovementioned dissolution media to recover all SNPs. The drug content was measured from standard calibration curves obtained analyzing a concentration range of 10–80 μg/mL, R^2 = 0.99 for P; 0.5–20 μg/mL for Q, R^2 = 0.98; 5–50 μg/mL for E, R^2 = 0.99. Data are reported as mean value ± standard deviation (n = 2).

In vitro drug release data confirmed what reported in the text of the manuscript. In EtOH, the percentage of drug released was 21.34 ± 5.639 for SNP-P, 50.69 ± 12.387 for SNP-Q and 63.35 ± 6.581 for SNP-E. In PBS, SNP-P and SNP-E released the 44.72 ± 8.593 and 48.27 ± 7.542 percentage of the whole drug, respectively, while SNP-Q released the 18.47 ± 6.522. After EtOH release, the amount of drug recovered after dissolution of SNPs was 73.22 ± 2.68 for SNP-P, 44.85 ± 5.986 for SNP-Q and 33.23 ± 4.896 for SNP-E. After PBS release, the amount of drug recovered after dissolving SNPs was 48.87 ± 2.569 for SNP-P, 75.58 ± 9.635 for SNP-Q and 47.58 ± 5.841 for SNP-E. Overall, the results confirmed that the drug not released was retained into SNPs.

References

1. Gaetani, P.; Torre, M.L.; Klinger, M.; Faustini, M.; Crovato, F.; Bucco, M.; Marazzi, M.; Chlapanidas, T.; Levi, D.; Tancioni, F., et al. Adipose-derived stem cell therapy for intervertebral disc regeneration: An in vitro reconstructed tissue in alginate capsules. *Tissue Engineering Part A* **2008**, *14*, 1415–1423, doi:10.1089/ten.tea.2007.0330.
2. Faustini, M.; Bucco, M.; Chlapanidas, T.; Lucconi, G.; Marazzi, M.; Tosca, M.C.; Gaetani, P.; Klinger, M.; Villani, S.; Ferretti, V.V., et al. Nonexpanded Mesenchymal Stem Cells for Regenerative Medicine: Yield in Stromal Vascular Fraction from Adipose Tissues. *Tissue Engineering Part C-Methods* **2010**, *16*, 1515–1521, doi:10.1089/ten.tec.2010.0214.
